# On the Probability Density of the Nuclei in a Vibrationally Excited Molecule

**DOI:** 10.3389/fchem.2019.00424

**Published:** 2019-06-06

**Authors:** Axel Schild

**Affiliations:** Laboratory for Physical Chemistry, ETH Zürich, Zurich, Switzerland

**Keywords:** molecular structure, normal modes, vibrational states, vibrational density, nuclear probability density, marginal density, one-body density, one-nucleus density

## Abstract

For localized and oriented vibrationally excited molecules, the qualitative features of the one-body probability density of the nuclei (one-nucleus density) are investigated. Like the familiar and widely used one-electron density that represents the probability of finding an electron at a given location in space, the one-nucleus density represents the probability of finding a nucleus at a given position in space independent of the location of the other nuclei and independent of their type. In contrast to the electrons, however, the nuclei are comparably localized. Due to this localization of the individual nuclei, the one-nucleus density provides a quantum-mechanical representation of the “chemical picture” of the molecule as an object that can largely be understood in a three-dimensional space, even though its full nuclear probability density is defined on the high-dimensional configuration space of all the nuclei. We study how the nodal structure of the wavefunctions of vibrationally excited states translates to the one-nucleus density. It is found that nodes do not necessarily lead to visible changes in the one-nucleus density: Already for relatively small molecules, only certain vibrational excitations change the one-nucleus density qualitatively compared to the ground state. It turns out that there are simple rules for predicting the shape of the one-nucleus density from the normal mode coordinates. A Python module for the computation of the one-nucleus density is provided at https://gitlab.com/axelschild/mQNMc

## 1. Introduction

Quantum-mechanically, a molecule is typically described with a complex-valued function Ψ, the wavefunction, that is a solution of the Schrödinger equation Ĥ_mol_Ψ = *E*Ψ with the molecular Hamiltonian Ĥ_mol_ that includes the Coulomb interactions among and between the electrons and nuclei of the molecule. If we want to understand features of the spatial molecular structure like the relative arrangement of the nuclei with respect to (w.r.t.) each other from the wavefunction, we would have to consider the corresponding probability density |Ψ|^2^ (in position space) that represent the probability of finding a particle within a certain volume of the configuration space of all particles. It follows that |Ψ|^2^ is a function which depends on the coordinates of all nuclei and electrons of the molecule and hence is a rather complicated object: Already for a small molecule like H_2_O, |Ψ|^2^ depends on the coordinates of thirteen particles (three nuclei and ten electrons) and is thus difficult to visualize, to comprehend, and also to measure.

Notwithstanding, in chemistry there exists an intuitive concept of a molecule. This concept is nicely illustrated by the idea of Lewis structures that indicate the participating compounds and the mechanism of a chemical reaction by means of graphs. The corners of the graphs are thought to represent the relative position of the nuclei (together with “core electrons”) in a three-dimensional space and the edges of the graph represent the electrons as “electron bonds.” The success of Lewis structures to encode and to rationalize chemical transformations suggests that somehow, from |Ψ|^2^ an effective three-dimensional picture of a molecule should emerge. We call this picture the “chemical picture” and understanding this emergence is a non-trivial and still partially open problem (Cafiero and Adamowicz, 2004, 2007; Sutcliffe and Woolley, 2005; Sutcliffe, 2010; Mátyus et al., 2011a,b; Mátyus and Reiher, 2012; Goli and Shahbazian, 2015) The aim of this paper is to investigate a certain aspect of the emergence: How the nuclei, whose quantum-mechanical probability densities depend on all their coordinates, become essentially points in a three-dimensional space in the chemical picture. This reduction of complexity can be achieved if the *N* nuclei are considered to be classical particles with their location being described by a point in the 3*N*-dimensional configuration space, which is equivalent to *N* points in a three-dimensional space. However, despite the success of this classical picture the nuclei are quantum particles, as is exemplified by recent interest in measuring e.g., the nuclear probability density (Shapiro, 1981; Zewail, 2000; Jurek et al., 2004; Ergler et al., 2006; Schmidt et al., 2012; Kimberg and Miron, 2014; Zeller et al., 2016) or the nuclear flux (current) density (Manz et al., 2013; Barth et al., 2015; Bredtmann et al., 2015). Hence, for the chemical picture to make sense it should be possible to obtain it quantum-mechanically without the need for (semi-)classical approximations.

In this article, we investigate how a three-dimensional nuclear structure emerges quantum-mechanically from |Ψ|^2^. As explained below, the equivalent quantum-mechanical operation of the reduction of a classical system from configuration space to a three-dimensional space is the calculation of the marginal probability density of a nucleus independent of its type, called the “one-nucleus density.” The nuclear (many-body) probability density in configuration space has often been calculated and analyzed (Smit et al., 2001; Dawes et al., 2013; Welsch and Manthe, 2015; Donoghue et al., 2016), but to this authors knowledge the one-nucleus has been calculated previously only for a few small molecules (Barth et al., 2008, 2009, 2012) and a systematic study of the one-nucleus densities of the eigenstates of a molecule is still missing. From the chemical picture, we expect that the one-nucleus density yields well-localized and spatially separated densities for each nucleus and that the localization depends on the nuclear mass. Additionally, the one-nucleus density should provide a quantum-mechanical picture of vibrational excitations: For classical nuclei, a vibrational excitation corresponds to a larger amplitude of motion, but quantum-mechanically the wavefunction of an excited state not only becomes more delocalized but also has nodes. The nodes exist in the configuration space of all nuclei but it is not obvious how they manifest in the one-nucleus density.

In the next sections, we discuss how the chemical picture of a molecule can be obtained conceptually as well as what the one-nucleus density is and how it is calculated. Thereafter, examples of the one-nucleus densities for vibrationally excited states of small molecules are given. It is shown how qualitative features of the one-nucleus density can be predicted from knowledge of the normal modes and it is discussed to what extend the idea that vibrationally excited nuclear states simply have a broader probability distribution than the vibrational ground state holds despite the nodes that such wavefunctions exhibit in the configuration space of all nuclei.

## 2. Theory

### 2.1. Obtaining the Chemical Picture of a Molecule

An important prerequisite for the obtaining the chemical picture of a molecule is the large mass difference of nuclei and electrons. Exact calculations for the static states of small systems show e.g., how the features of the molecular wavefunction |Ψ|^2^ change when H^−^ becomes ${\text{H}}_{2}^{+}$, which is mathematically “just” a change of the mass of one particle (|Ψ|^2^ is invariant w.r.t. charge conjugation) (Mátyus et al., 2011a,b; Mátyus and Reiher, 2012). The mass difference motivates a separation of the molecular wavefunction into a (marginal) nuclear wavefunction and an electronic wavefunction that conditionally depends on the location of the nuclei,

(1) $$\begin{array}{ll} \Psi \left(X,x\right) & =\psi_{\text{nuc}}\left(X\right)\psi_{\text{el}}\left(x|X\right), \end{array}$$

where *X* and *x* represent the coordinates of all nuclei and electrons, respectively. While such a separation is exact and can be used to define a nuclear wavefunction (Hunter, 1975; Abedi et al., 2010, 2012), its practical application is usually in terms of the Born-Oppenheimer approximation (Born and Oppenheimer, 1927; Eich and Agostini, 2016) where the effect of the nuclear motion on the electronic wavefunction is neglected and only the chosen positions of the nuclei are relevant (Schild et al., 2016).

However, the nuclear probability density $|\psi_{\text{nuc}}{|}^{2}$ of a single water molecule still depends on nine coordinates and thus does not directly correspond to the corners of the Lewis structure that are thought to represent the relative position of the nuclei w.r.t. each other. There are two additional problems when the molecule is assumed to be a closed system: The first problem is that for a perfectly isolated molecule the density $|\psi_{\text{nuc}}{|}^{2}$ would be a constant, because only relative interactions exists and the Hamiltonian is invariant w.r.t. translation of the whole system. This view of an isolated molecule is a bit useless but can be remedied by taking a relative view. A sensible choice would be to give up the idea of considering the molecule as a closed system and to define its coordinates relative to an external system. This choice is discussed further below. What is typically done instead is to chose an internal coordinate to which the coordinates of the nuclei are referred to, in particular the center-of-mass coordinates of either the nuclei or of the whole molecule. Then the nuclear wavefunction is

(2) $$\begin{array}{l} \psi_{\text{nuc}}\left(X\right)=\psi_{\text{nuc}}^{\text{com}}\left(X_{\text{com}}\right)\psi_{\text{nuc}}^{\text{rel}}\left(X_{\text{rel}}|X_{\text{com}}\right), \end{array}$$

where $\psi_{\text{nuc}}^{\text{com}}$ is the wavefunction of the center of mass with the (three) coordinates *X*_com_ and $\psi_{\text{nuc}}^{\text{rel}}$ is the wavefunction of the relative coordinates *X*_rel_ that conditionally depends on *X*_com_. For a closed system without external interactions this conditional dependence vanishes (for the center of mass of the molecule or, in the Born-Oppenheimer approximation, for the center of mass of the nuclei) and $\psi_{\text{nuc}}^{\text{rel}}\left(X_{\text{rel}}|X_{\text{com}}\right)\equiv \psi_{\text{nuc}}^{\text{rel}}\left(X_{\text{rel}}\right)$ can be obtained independent of *X*_com_.

The second problem when the molecule is considered to be a closed system is that the probability density $|\psi_{\text{nuc}}^{\text{rel}}\left(X_{\text{rel}}\right){|}^{2}$ after separation of the center of mass is spherically symmetric because the Hamiltonian is invariant w.r.t. rotation of the whole molecule (Mátyus et al., 2011a,b; Mátyus and Reiher, 2012). Such a bubble-shaped density has interesting dynamical features (Manz et al., 2014; Bredtmann et al., 2015; Pérez-Torres, 2015; Diestler et al., 2018) and is an excellent approximation to the rotational eigenstates of single molecules in vacuum, but it is of limited help to understand how Lewis structures represent a molecule. The wavefunction $\psi_{\text{nuc}}^{\text{rel}}$ can, however, approximately be written as (Bunker and Jensen, 2006)

(3) $$\begin{array}{l} \psi_{\text{nuc}}^{\text{rel}}\approx \psi_{\text{nuc}}^{\text{rot}}\times \psi_{\text{nuc}}^{\text{vib}}. \end{array}$$

where $\psi_{\text{nuc}}^{\text{rot}}$ depends on three coordinates that describe the overall rotation of the molecule and $\psi_{\text{nuc}}^{\text{vib}}$ may be interpreted as describing the vibrations of the molecule. For water, $|\psi_{\text{nuc}}^{\text{vib}}{|}^{2}$ depends on three coordinates, but for *N* nuclei $|\psi_{\text{nuc}}^{\text{vib}}{|}^{2}$ depends on 3*N* − 6 coordinates (or 3*N* − 5 coordinates for linear nuclear configurations) and describes the shape of the molecule in configuration space for a given location and orientation. Thus, it encodes the relative location of the nuclei w.r.t. each other but it is not the three-dimensional arrangement that the corners of a Lewis structure are representing.

From the preceding discussion follows that for the chemical picture, it is necessary to have a localized and oriented the molecule so that we can go back to the full nuclear wavefunction ψ_nuc_(*X*) and determine reduced quantities that yield the information about the nuclear structure that we are interested in, i.e., the one-nucleus density. Before doing that by simply assuming a distribution such that the molecule is localized and oriented, we would like to comment on how the one-nucleus density could be obtained more rigorously.

The localization and orientation of the molecule can be achieved conceptually by changing from an absolute view of a molecule to a relative view where there is a second system which the molecule is related to. We might call the second system an “environment.” This environment can e.g., be other molecules (like in a liquid phase or during a chemical reaction), a surface with which the molecule is interacting, or a suitably chosen laser pulse. We can then obtain a Schrödinger equation for the molecule alone by either choosing it as a marginal system (like a Schrödinger equation can be obtained for the nuclei in a molecule alone, which is, however, representing the whole molecule Hunter, 1975; Abedi et al., 2010, 2012) or as a system that conditionally depends on the state of a second system (which yields a generalization of the time-dependent Schrödinger equation Schild, 2018). In any case, we may remove the translational and rotational degrees of freedom of the combined system and work within a relative picture. The interaction with the environment can then result in a localization and orientation of the molecule.

Following this procedure to embed the molecule in an environment is mathematically challenging and depends on the actual problem under study. It will not be done or needed in this article but is outlined here to motivate why we will, in the following, use calculations for isolated molecules but still assume that the molecule is located and oriented (relative to another molecule, a surface, etc.). Clearly, the interaction with an environment changes the problem and thus also the details of the nuclear densities that are presented below. Nevertheless, the general rules given below for predicting the shapes of the one-nucleus densities still apply but care has to be taken to determine the normal modes correctly.

### 2.2. The One-Nucleus Density

In the theoretical description of a molecule, the nuclei are often treated as (semi-)classical particles with a definite configuration and momentum. In contrast, the electrons are usually treated quantum-mechanically and the methods of Quantum Chemistry are used to approximately determine their wavefunction (Szabo and Ostlund, 1996). The corresponding many-electron probability density depends on the coordinates of all electrons and, although the correlated two-electron probability density of the H_2_ molecule was measured (Waitz et al., 2017), for more electrons it is a rather inaccessible observable. An alternative quantum-mechanical observable that is defined in a three-dimensional space is the electronic one-body probability density, also known as the one-electron density. The one-electron density is the marginal density of finding one electron at a given location in space independent of where the other electrons are. It is the central quantity of Density Functional Theory (Ullrich, 2012), it is easily visualized, and it is directly accessible to experiment if the molecule is localized: For example, an electron scanning tunneling microscope does essentially measure the one-electron density of a localized molecule and can provide intuitive images of molecules on surfaces (Moore and Weiss, 2008). However, while some information about the electronic state can be extracted from the one-electron density (Baer, 2010), this task is in general difficult: Although the many-electron density is qualitatively different for different excited states due to the appearance of nodes in the wavefunction, the corresponding one-electron densities may be very similar. That the information content of the one-electron density is comparably limited (in the sense that it is hard to extract; formally, it contains similar information like the many-electron density) can be explained by the rather delocalized many-electron density. The one-electron density is obtained by integration of the many-electron density over all but the coordinates of one electron, and delocalization means that there is a relatively large part of the many-electron configuration space which contributes to the integral. In an excited electronic state sign-changes in the many-electron wavefunction lead to a depletion and nodes of the many-electron density in some regions of configuration space compared to the ground state, but due to the integration which also includes (approximately) unchanged regions of configuration space, the one-electron density is likely to be little affected. This is also one of the reasons why, instead of the one-electron density, electron orbitals as effective single-electron wavefunctions are needed in chemistry to rationalize many chemical reaction mechanisms. Electron orbitals are three-dimensional mathematical objects that are rather intuitive and that encode important information about the full electronic wavefunction which is not easily accessible in the one-electron density. Also, it may be argued that they are effectively present in the Lewis structures in the form of “electron bonds” indicated by the connections in the graphs.

The situation is different for the nuclei because, due to their large mass, the nuclear wavefunction is rather localized in some regions of the nuclear configuration space. This raises the question if the analog of the one-electron density for the nuclei, the one-nucleus density, contains accessible information about the nuclear wavefunction. In particular, we want to investigate if it provides information about the vibrational state of the molecule, i.e., would the nodal structure of wavefunctions in excited states be visible in the one-nucleus density? This question is answered in the following with the help of the nuclear wavefunction obtained from a normal mode analysis, i.e., obtained from the local harmonic approximation of the potential energy surface for the nuclear configuration of lowest energy (Henriksen and Hansen, 2011). The nuclear wavefunction ψ_nuc_ is then a product of a translational, a rotational, and a vibrational part,

(4) $$\begin{array}{l} \psi_{\text{nuc}}=\psi_{\text{nuc}}^{\text{com}}\times \psi_{\text{nuc}}^{\text{rot}}\times \psi_{\text{nuc}}^{\text{vib}}. \end{array}$$

The translational part $\psi_{\text{nuc}}^{\text{com}}$ represents translation of the center of mass of the nuclei in space and can, within the Born-Oppenheimer approximation, be factored exactly, while the separation of $\psi_{\text{nuc}}^{\text{rot}}$ (which describes rotations of the whole molecule) from $\psi_{\text{nuc}}^{\text{vib}}$ is only valid for small displacements from the equilibrium configuration (Eckart, 1935; Littlejohn and Reinsch, 1997; Bunker and Jensen, 2006; Lauvergnat et al., 2016). Typically, a normal mode analysis aims at computing the vibrational frequencies (and maybe analyzing the normal mode coordinates) while ψ_nuc_ is of little interest. However, if those frequencies are in good agreement with measured frequencies, the function ψ_nuc_ is likely also a good approximation to the exact nuclear wavefunction. Then, statements about how qualitative features of ψ_nuc_ translate to the approximate one-nucleus density can be expected to be also true for the exact one-nucleus density. Notwithstanding, anharmonic effects may be important and could potentially even change the qualitative outcome, as discussed below.

In the following, the one-nucleus densities of the nuclei for different states of $\psi_{\text{nuc}}^{\text{vib}}$ are studied and it is investigated how nodes of the wavefunction in excited states manifest in the one-nucleus density. In analogy to the classical representation of the *N* nuclei as *N* points in a three-dimensional space, the sum of the one-nucleus densities for all individual (types of) nuclei yields a density in three-dimensional space where, in contrast to the one-electron density, the probability distributions of each nucleus are well-separated and clearly visible. For brevity, hereafter this sum is simply called the one-nucleus density (in analogy to the one-electron density) and the nuclear many-body probability density in the *N*-dimensional configuration space is called the *N*-nucleus density. A detailed description of how the one-nucleus densities are obtained is given in the Supporting Information. Here, only the approximations and assumptions are discussed to point out when the approach is applicable. The aim is to determine the one-nucleus density $\rho \left(\vec{R}\right)$ from the approximate nuclear wavefunction ψ_nuc_(*X*), where $\vec{R}=\left(R_{1},R_{2},R_{3}\right)$ is a three-component vector and where $X=\left({\vec{X}}_{1},\ldots ,{\vec{X}}_{N}\right)$ stands for the *N* three-component position vectors ${\vec{X}}_{j}$ of the nuclei. The one-nucleus densities for each nucleus are obtained by integrating the *N*-nucleus density $|\psi_{\text{nuc}}\left(X\right){|}^{2}$ over all but the coordinates of the selected nucleus,

(5) $$\begin{array}{l} \rho_{j}\left(\vec{R}\right)=\int \cdots \int |\psi_{\text{nuc}}\left(X\right){|}^{2}d{\vec{X}}_{\left\{1\cdots N_{n}\right\}\backslash j}{|}_{{\vec{X}}_{j}=\vec{R}} \end{array}$$

for $d{\vec{X}}_{\left\{1\cdots N_{n}\right\}\backslash j}=d{\vec{X}}_{1}\ldots d{\vec{X}}_{j-1}d{\vec{X}}_{j+1}\ldots d{\vec{X}}_{N_{n}}$. The approximate nuclear wavefunction that we use does not obey any exchange symmetry for identical nuclei, hence ρ_*j*_ needs to be computed for each single nucleus and not only once for identical nuclei. The neglect of exchange symmetry is not a problem for the systems studied here because the nuclei are localized and there is a large energy barrier for exchanging nuclei. Hence the one-nucleus density of the molecule, which we define as the sum of the one-nucleus densities for all nuclei,

(6) $$\begin{array}{ll} \rho \left(\vec{R}\right) & =\overset{N}{\underset{j=1}{\sum }}\rho_{j}\left(\vec{R}\right), \end{array}$$

is (for the cases discussed below) practically identical to the one obtained from the corresponding wavefunction with correct exchange symmetry. The meaning of the one-nucleus density given in (6) is that it is the probability to find any nucleus in a given region of space independent of its type. However, from the relative spatial location it is immediately clear if the nucleus is e.g., an oxygen or a hydrogen nucleus.

The nuclear wavefunction is obtained as follows: (1) The Born-Oppenheimer approximation is made to obtain a Schrödinger equation for the nuclear wavefunction alone, with a potential energy surface *V*(*X*). Only the electronic ground state is considered. (2) A standard normal mode analysis (Henriksen and Hansen, 2011) at one of the minima of *V* is made. From this calculation *N* normal mode coordinates *q*_*j*_ and the frequencies of their harmonic oscillator (HO) potentials are obtained. The nuclear wavefunction is a product of HO wavefunctions in each normal mode coordinate, and coupling of rotational and vibrational degrees of freedom is neglected (Littlejohn and Reinsch, 1997; Bunker and Jensen, 2006). (3) There are six (five for linear molecules) normal modes for which the corresponding HO frequencies are zero, representing translation and rotation of the molecule. The nuclear wavefunction has the form of (4). It is assumed that $\psi_{\text{nuc}}^{\text{com}}$ and $\psi_{\text{nuc}}^{\text{rot}}$ are normalized Gaussian functions with a very small width corresponding to a frequency of 0.5 *E*_*h*_/ℏ. This acts like a constraint on the coordinates and has the effect of localizing and orienting the molecule. The resulting probability density can be interpreted either as a cut through the full density or as the nuclear density given the molecule is at a certain position and oriented in a certain way. A practical advantage of this choice for $\psi_{\text{nuc}}^{\text{com}}$ and $\psi_{\text{nuc}}^{\text{rot}}$ is that the wavefunction becomes a product of HO eigenfunctions in all modes. The nuclear wavefunction is

(7) $$\begin{array}{l} \psi_{\text{nuc}}\left(X\right)=\overset{3N}{\underset{j=1}{\prod }}\phi_{j}\left(q_{j}\left(X\right),n_{\nu_{j}}\right), \end{array}$$

where ϕ_*j*_(*q*_*j*_, *n*_ν_*j*__) is a HO wavefunction with quantum number *n*_ν_*j*__ for mode ν_*j*_, and where *n*_ν_*j*__ = 0 for the normal mode coordinates for translation and rotation of the whole molecule. In the Supporting Information, it is described how the high-dimensional integral of (5) with wavefunction (7) can be solved analytically. All quantum chemical calculations are made with the program Psi4 (Turney et al., 2012) using 3rd order Møller-Plesset perturbation theory and a cc-pVTZ basis set (Dunning, 1989). This level of electronic structure theory is suitable for the molecules presented in this article because it reproduces the experimentally known vibrational frequencies reasonably well and because the results shown here do not depend critically on the accuracy of the harmonic approximation. However, there can be cases where and accurate description of the potential energy surface is more important, as discussed below.

### 2.3. Implementation: The mQNMc Python Module

For the calculation of the one-nucleus density the Python 3 module mQNMc was developed. This module is available at https://gitlab.com/axelschild/mQNMc and is released under the GNU General Public License version 3. It uses the output of a Psi4 calculation as input, but adding interfaces to other programs is straightforward because mQNMc needs only the Hessian matrix, the nuclear configuration and the nuclear masses to perform the calculation. The codes for generating the figures of the water molecule are given as examples in the module and can be used as a template for other calculations.

## 3. Results

**Figure 1:**
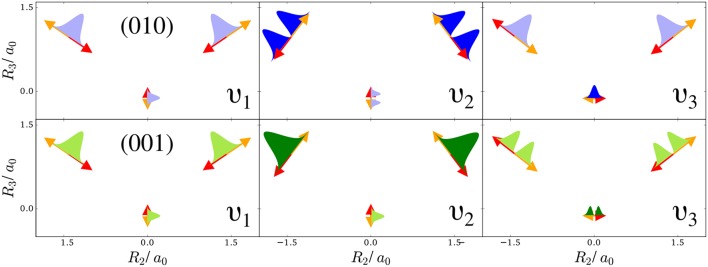
Normal modes of a water molecule in the molecular plane (arrows show the extent and directionality (color) of the nuclear displacement along the mode): Symmetric stretch ν_1_, bending mode ν_2_, and antisymmetric stretch ν_3_. The top-row shows sketches of the harmonic oscillator densities along the modes for the first excitation of the bending mode (010), the bottom row for the first excitation of the antisymmetric stretch (001). A light filling of these densities indicates that locally other modes point in the same direction, while a dark filling means that this is not the case.

**Figure 2:**
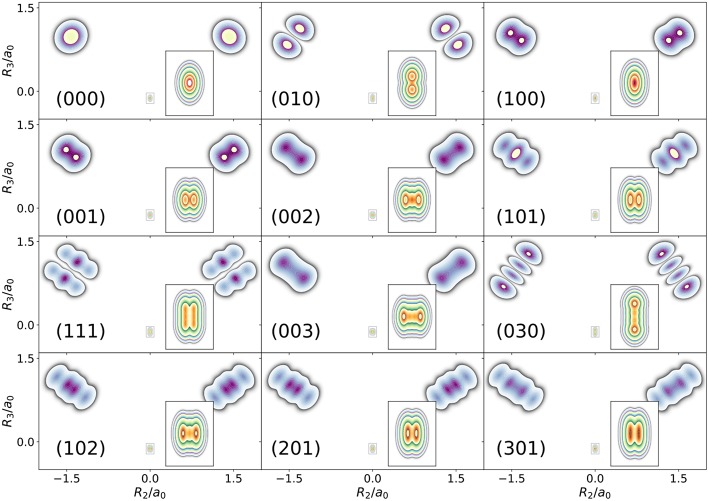
Contour plots of the one-nucleus density of a localized and oriented water molecule in the molecular plane for different vibrationally excited states. State labels (*n*_ν_1__, *n*_ν_2__, *n*_ν_3__) indicate the number of quanta in normal modes ν_1_, ν_2_, ν_3_, respectively.

The first example is the one-nucleus density of the water molecule. Figure 1 shows its familiar vibrations as arrows indicating the motion of classical nuclei. There are three vibrational normal mode coordinates which are labeled according to the usual spectroscopic notation (Shimanouchi, 1972): the symmetric stretch ν_1_, the bending mode (scissoring) ν_2_, and the antisymmetric stretch ν_3_. The one-nucleus densities of the water molecule for some excitations of these modes are shown in Figure 2 as contour plots in the molecular plane, labeled as (*n*_ν_1__, *n*_ν_2__, *n*_ν_3__), where *n*_ν_*j*__ is the quantum number of mode ν_*j*_. Insets magnify the details of the nuclear density around the oxygen nucleus, as the density there is much more localized compared to the hydrogen nuclei due to the mass difference.

The one-nucleus density of the vibrational ground state (000) shown in Figure 2 looks for each nucleus like a product of Gaussian functions oriented along the directions of the normal modes. The one-nucleus densities of the first excitation in each mode, (100), (010), and (001), show how a node in the first excited state of the HO along the corresponding normal modes translates to the one-nucleus density. From the pictures, it seems that the node in the wavefunction of one of these modes leads to a depletion of the one-nucleus density along this normal mode coordinate, but not to exact nodes or nodal planes in the one-nucleus density. There are two reasons for the absence of exact nodes: First, a Gaussian distribution for the translational and rotational modes is assumed. Depending on the width of the these distributions, the resulting one-nucleus densities become broader and loose their structure. A very narrow Gaussian distribution is chosen, hence this reason is of minor importance. The main reasons for the absence of exact nodes in the one-nucleus density is that those nodes only exists in configuration space, while the reduction of the *N*-nucleus density to the one-nucleus density as given in (5) in general does not yield zero anywhere in space.

To understand the one-nucleus density in Figure 2, the analytic form of the *N*-nucleus density needs to be investigated. It is a product of HO densities in all normal modes, because the wavefunction (7) is a product of HO wavefunctions. These 1d-HO densities can be visualized by functions centered at the equilibrium position of the three nuclei, with extent and direction as given by the arrows. In Figure 1, the idea is illustrated for the first excited state of the bending mode, (010), and of the antisymmetric stretch, (001).

Some predictions can be made about the qualitative features of the one-nucleus densities at the nuclei by means of a set of simple rules. These rules are called the LOcal COmparison (LOCO) rules, because they are based on a comparison of the normal mode coordinates at the location of each nucleus separately. The LOCO rules are as follows: For a nucleus, the magnitudes and directions of the displacements along the normal modes are compared. (a) If only one normal mode displaces the nucleus in a certain direction or if there is one normal mode that displaces the nucleus in a certain direction much stronger than the other normal modes, the nodes of the wavefunction due to an excitation of this normal mode are clearly visible as depletions in the one-nucleus density. (b) If several normal modes displace the nucleus in the same direction by similar magnitude, an excitation of one of these modes is not necessarily visible in the one-nucleus density. In general, the more such modes exist, the less likely it is that an excitation in one of these can be recognized in the one-nucleus density. It follows that typically, only the normal modes that displace a nucleus the most in a given direction can have a strong influence on the qualitative shape of the one-nucleus density. (c) If there are two (or more) modes that displace the nucleus in the same direction, simultaneous excitation of these modes may show combination features, as exemplified below.

For example, the one-nucleus density of states (100), (010), and (001) can be understood from the LOCO rules (a) and (b) as follows: In Figure 1, sketches of the harmonic oscillator wavefunctions in the modes are shown. At the hydrogen nuclei, ν_1_ and ν_3_ point in a similar direction, while ν_2_ is perpendicular. Thus, the excitation of ν_2_ state (010) leads almost to a nodal plane in the one-nucleus density at the hydrogen nuclei, cf. Figure 2. In contrast, excitation of ν_1_ state (100) or ν_3_ state (001) lead to a significantly less pronounced depletion of the one-nucleus density at the equilibrium position of hydrogen. For the oxygen nucleus, the situation is reversed, as ν_1_ and ν_2_ point in the same direction and ν_3_ is perpendicular. Consequently, the one-nucleus density of state (001) shows a depletion at the oxygen equilibrium position, while no depletion is seen in state (100). As ν_2_ displaces the oxygen nucleus stronger than ν_1_ (which is, however, hardly visible on the scale of Figure 1), a depletion due to the node in ν_2_ is visible in state (010).

A situation similar to that of state (001) is found for states (002) and (003), i.e., when the HO function of ν_3_ is in its second or third excited state. For (002), there is the expected triple-maximum structure at the oxygen nucleus (with a lower central maximum), while the central maximum at the hydrogen nuclei is not visible. For state (003) four maxima are found in the one-nucleus density at the oxygen nucleus (although the two central ones are too weak to be clearly seen in Figure 2), while at the hydrogen nuclei still only two maxima can be found.

An example for LOCO rule (c) is found if two normal modes are excited that locally have similar magnitude and direction. For state (101) three maxima appear at the hydrogen nuclei, similar to a second excited state of the HO, but the central maximum is strongest while the outer maxima are weaker. At the oxygen nucleus only two maxima that point along the direction of normal ν_3_ are found, i.e., a combination of the features of the one-nucleus densities for states (100) (only one maximum in the direction of ν_1_) and (001) (two maxima in the direction of ν_3_). For states (102) and (201) the qualitative features of the one-nucleus densities can be explained analogously and in accord with the LOCO rules, especially the four maxima at the hydrogen nuclei and a triple maximum at the oxygen nucleus for state (102), but only a double maximum at the state (201).

Last, for state (111) the resulting one-nucleus density is a simple combination of features of states (010) and (101), because ν_2_ is locally perpendicular to the other two vibrational modes at the hydrogen nuclei. At the oxygen nucleus, the effect of exciting two modes with similar direction and magnitude, LOCO rule (c), is that three maxima are found along coordinate *R*_3_ (as defined in the figure) upon closer inspection, although the central one is hardly visible.

**Figure 3:**
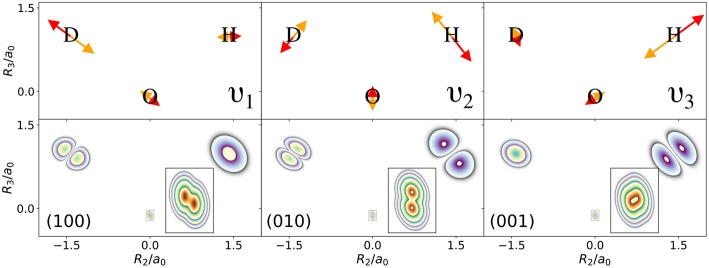
(Top) Normal mode coordinates of the mono-deuterated water molecule in the molecular plane: O-D stretch ν_1_, bending mode ν_2_, O-H stretch ν_3_. (Bottom) Contour plots of the one-nucleus density of a localized and oriented mono-deuterated water molecule in the molecular plane for vibrational states corresponding to the first excitations of the normal modes shown above.

The normal modes of the water molecule have a symmetry with respect to the hydrogen nuclei. If the symmetry of the nuclear structure is broken by replacing one hydrogen nucleus with a deuterium nucleus, a very different picture for the one-nucleus densities is obtained. The normal mode coordinates for a mono-deuterated water molecule are given in Figure 3. The bending mode ν_2_ is similar to the bending mode of water, but ν_1_ is the O-D stretch mode that only displaces the deuterium nucleus strongly, while ν_3_ is the O-H stretch mode that almost exclusively displaces the hydrogen nucleus. Thus, according to the LOCO rules it is expected that any excitation of ν_2_ is clearly visible in the one-nucleus density at the hydrogen and deuterium nucleus, that an excitation of ν_1_ is visible at the deuterium nucleus but not at the hydrogen nucleus, and that an excitation of ν_3_ is visible at the hydrogen nucleus but not at the deuterium nucleus. The prediction of the LOCO rules is accurate, as the one-nucleus densities of the first excited states of each of these modes shown in Figure 3 illustrate.

A consequence of the LOCO rules is that for molecules with many nuclei, only certain (of the lowest) excited states are qualitative visible in the one-nucleus density, i.e., those that are the ones displacing a nucleus the most in a certain direction of space. This is indeed the case. For example, for the benzene molecule the one-nucleus density of none of the first excited states of any normal mode in the molecular plane is qualitatively different from the vibrational ground state. The situation changes if one hydrogen nucleus is exchanged by a deuterium nucleus. In the Supporting Information, the one-nucleus densities for the only two normal mode coordinates that strongly displace the deuterium nucleus are shown. Further examples given in the Supporting Information are an analysis of ethene and mono-deuterated ethene as molecules that contain less nuclei than benzene but where similar effects are found, and methane as an example of a non-planar molecule.

## 4. Conclusion

The discussed examples of the one-nucleus densities show that an important part of the chemical picture of a molecule can also be obtained within a quantum-mechanical treatment: Except for light quantum nuclei like hydrogen or deuterium, the one-nucleus density is very localized around the equilibrium position of the nuclei. Hence, it yields a three-dimensional relative arrangement of the nuclei that is similar to what is expected for a classical approximation of the nuclear wavefunction. It is interesting to compare this result with the similar three-dimensional representation of quantum nuclei by means of path integral methods, where the density matrix for finite temperatures is written as a path integral that can be interpreted as an integral over a classical system composed of “a chain of beads connected with springs” for each nucleus (Ceperley, 1995). The path-integral formulation also yields a three-dimensional representation of the nuclear density that includes quantum effects (see e.g.,Richardson, 2018) but that does not correspond to any observable, whereas the one-nucleus density is an observable that can in principle be measured.

Importantly, in contrast to the one-electron density where the electronic state is in general not qualitatively visible (or where, to this authors knowledge, there are no rules available to predict if this is the case), the vibrational state of a molecule may be directly visible in the one-nucleus density. The presented LOCO rules allow to predict, from a normal mode analysis of the nuclear wavefunction, without much effort which vibrational excitations would be visible in the one-nucleus density. From the LOCO rules follows that in many case the classical idea that vibrationally excited molecules simply have a larger amplitude of vibration is in fact also what the one-nucleus density shows. Only for small molecules or certain vibrational modes the excitation can clearly be seen as qualitative change compared to the ground state.

The description of the potential energy surface can be important for the one-nucleus density, because the LOCO rules show that the relative amplitude of the normal modes matters. This relative amplitude has to be described accurately with a suitable electronic structure method. In this respect, the underlying harmonic approximation of the potential is a limitation of the model. Anharmonic effects will for many molecules not change the LOCO rules, but even for molecules that have no large-amplitude mode they can be important for the relative amplitudes of the different vibrations and thus for the influence that a certain mode has on the one-nucleus density. However, an extension of the harmonic model to include anharmonic effects is problematic because the advantage that the integrals can be treated analytically may be lost. A numerical evaluation of the high-dimensional becomes infeasible already for small molecules, hence analytic solutions are highly desirable.

Despite the limitations of the presented analysis, a careful application of the LOCO rules together with the harmonic approximation can in principle be used to guide a possible experimental study: A suitable target molecule and target state for which the one-nucleus density might be measurable can be found, and the Python module mQNMc can be a useful for that purpose. Although it is difficult to measure details of a very localized density, the many-body nuclear density is a prohibitively complicated object for larger molecules because all electrons have to be measured in coincidence and because the data is hard to interpret. Thus, the one-nucleus density can be an interesting alternative observable to learn more about the quantum nature of molecular structure.

## Data Availability

The raw data supporting the conclusions of this manuscript will be made available by the authors, without undue reservation, to any qualified researcher.

## Author Contributions

AS designed and carried out the research, the calculations, and the numerical implementation, and wrote the article.

### Conflict of Interest Statement

The author declares that the research was conducted in the absence of any commercial or financial relationships that could be construed as a potential conflict of interest.
