# Supplementary material for: On the Probability Density of the Nuclei in a Vibrationally Excited Molecule
Source: Front Chem. 2019 Jun 6;7:424. doi: 10.3389/fchem.2019.00424 (PMC6562893; doi:10.3389/fchem.2019.00424)
Supplement: Supplementary file 1 [file Data_Sheet_1.PDF]

Supporting Information for "On the Probability Density of the Nuclei in a Vibrationally Excited Molecule": Additional Examples

Axel Schild

March 1, 2019

A note on the labeling of the normal modes: *In contrast to the main article, molecules with more that three nuclei are discussed in the following, hence there are more normal modes. To have a uniform and simple notation for all molecules, the following convention is used to label the normal modes: The normal modes are numbered according to the frequency of their corresponding harmonic oscillators in ascending order. Modes 1 to 6 are those of translation and rotation of the molecule. The symmetry of the mode is ignored in the numbering.*

A note on the labeling of the states: *In these document, only one-nucleus densities for a single excitation of one mode or, for methane, for two singly excited modes or one double excited mode are shown. The state is labeled by the number(s) of the excited modes.*

In the following, present one-nucleus densities for selected states of water, mono-deterated benzene, ethene, mono-deuterated ethene (D-ethene), and methane are presented and it is discussed how their qualitative shape can be predicted by using the LOCO rules and the normal mode coordinates. All computations were performed as described in the main article, hence the Born-Oppenheimer approximation is used in a local harmonic approximation at the nuclear configuration of minimum energy (the equilibrium configuration), and it is assumed that the molecule is localized and oriented. The three-dimensional reference space has coordinates $\mathtt{R}_1, \mathtt{R}_2, \mathtt{R}_3$. For each molecule, first the normal mode coordinates are discussed and thereafter selected densities are presented.

As mentioned in the main article, for the benzene molecule the first excitations of any of the normal mode coordinates do not lead to a qualitative change of the one-nucleus density compared to the ground state because the hydrogen and oxygen nuclei are displaced in the same direction by multiple normal modes. In contrast, for mono-deterated benzene there are only two normal modes that significantly displaced the deuterium nucleus. These coordinates are also perpendicular with respect to each other, hence their excitations are clearly visible in the one-nucleus density. This is shown in figure 1.

The equilibrium configuration of ethene is planar, hence the normal mode coordinates correspond to displacements of the nuclei that are either completely in the molecular plane, or perpendicular. Hence, the discussion is restricted to the normal modes in the molecular plane, which is defined as the $\mathtt{R}_2$-$\mathtt{R}_2$ plane. For ethene, figure 2 shows the selected normal mode coordinates. The modes are numbered by increasing frequency of the corresponding harmonic oscillator, and modes 1-6 are those of translation and rotation of the whole system.

From the figure, it can be seen that at the hydrogen nuclei there are many normal modes that correspond to displacements in a similar spatial direction, which also have similar magnitude. For example, modes 7, 11, 12, and 13 all displace the hydrogen nuclei to a similar extend in a similar direction, while modes 15, 16, 17, and 18 do the same in an almost perpendicular direction. Hence, from the LOCO rules it can be concluded that an excitation of just one of these modes does not yield any notable qualitative changes of the one-nucleus density, i.e. there are no new clear minima appearing that correspond to the nodes of the wavefunction in such an excited state. This is indeed the case, and all one-nucleus densities for an excitation along one of these modes is qualitatively similar to the ground state.

The situation at the carbon nuclei, however, is somewhat different. Although there are again many modes that displace these nuclei in the same direction, there are two modes that displace them stronger than all other modes: Mode 11 displaces the oxygen nuclei comparably strongly along $\mathtt{R}_2$, while mode 14 displaces the oxygen nuclei comparably strongly along $\mathtt{R}_3$. This difference can be seen in the one-nucleus densities for the respective excited states. Figure 3 shows contour plots of the one-nucleus densities for the first excited states of mode 11 and mode 14, and insets show a magnification of the regions around the carbon nuclei. Several contour maps with different line spacing are used to be able to see details of the density of the hydrogen nuclei and of the carbon nuclei in the same picture. The density at those nuclei has two local maxima and a depletion in the region of the equilibrium position. No other of the excited states corresponding to the first excitation any of the normal modes shows this qualitative features, neither at the carbon nuclei nor at the hydrogen nuclei.

The situation is altered if the symmetry is broken by isotope substitution. The normal mode coordinates for D-ethene are given in figure 4. As is clear from the figure, there are several normal modes that should, if excited, according to the LOCO rules yield a clear qualitative imprint in the one-nucleus density, because they are the only ones that

displace the considered nucleus significantly in a certain direction. To illustrate, normal modes 7, 12, 15, and 17 are used. Normal modes 7 and 15 are the ones that displace the deuterium nucleus the strongest, and in almost perpendicular directions. Modes 12 and 17 displace the top-right hydrogen nucleus the strongest, and also in mutually almost perpendicular directions. Consequently, excitations of those modes can be expected to have the strongest qualitative impact on the one-nucleus density at the given nucleus.

Figure 5 shows the one-nucleus density for the excites states represented by first excitation of modes 7, 12, 15, or 17. It can clearly be seen that excitations of mode 7 and 15 as well as 12 and 17 show two maxima and a depletion corresponding to the node in the wavefunction of the excited state, at the deuterium nucleus and the top-right hydrogen nucleus, respectively. There are more examples of this behavior at the other nuclei, but none of these is as pronounced as the ones depicted in figure 5.

Last, it should be illustrated that the LOCO rules also hold for non-planar molecules. For this purpose, methane is considered. In its equilibrium configuration, this molecule has four hydrogen nuclei at the vertices of a tetrahedron, and a carbon nucleus at its center. As it is hard to draw the one-nucleus density of the nuclei in a picture, only one of the four equivalent hydrogen nuclei is considered. For the carbon nucleus being at the origin, this nucleus is located at ca. $(0, 2, 0)a_0$. All normal mode coordinates at this nucleus are shown in figure 6 as arrows in the R_1-R_2-, R_1-R_3-, and R_2-R_3-plane.

From the figure, it can be seen that at this nucleus, in each direction there are only two relevant normal modes: mode 8 and 10 along R_1, mode 12 and 15 along R_2, and mode 9 and 11 along R_3. Mode 8, 15, and 9 are those displacing the nucleus the strongest, although only mode 15 has a clear margin with respect to mode 12, while the displacement along modes 10 and 11 are very close to those of modes 8 and 9, respectively

In this example, the excited states corresponding to two quanta in these modes are considered. Contour plots of the one-nucleus densities are given in figure 7 for excitations of mode 8 and 10, in figure 8 for excitations of mode 12 and 15, and in figure 9 for excitations of mode 9 and 11.

It is found that a double excitation of mode 15 has a clear triple-maximum structure reminiscent of the density of the harmonic oscillator wavefunction in this node. According to the LOCO rules this is expected, because mode 15 displaces the considered nucleus the most. Also double excitations of modes 8 or 9 yield triple-maximum structures, although the central maximum is almost invisible. Again, this can be rationalized because the wavefunction is in its ground state along modes 10 and 11, which have similar effects on the nucleus.

The combined effect of exciting two locally similar modes can also be observed. In figures 7, 8, and 9, the one-nucleus density of the excited state corresponding to an excitation of mode 8 and 10, mode 12 and 15, and mode 9 and 11 are also shown. A triple-maximum structure is found, which is most pronounced when the two modes are locally similar, i.e. for excitation of mode 8 and 10 as well as mode 9 and 11.

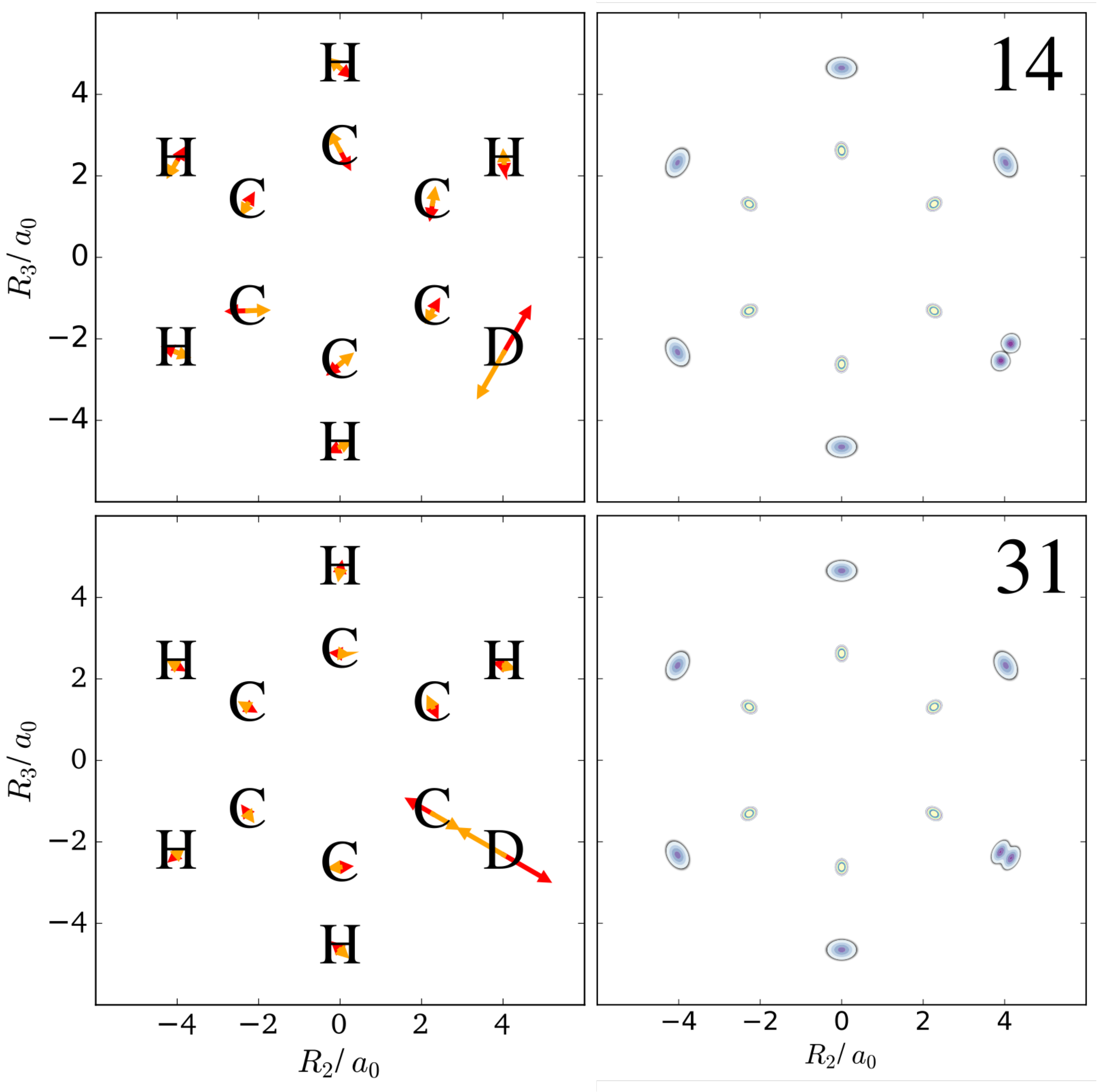

Figure 1: Left: Normal mode coordinates of the mono-deuterated benzene molecule. Right: Contour plots of the one-nucleus densities of a localized and oriented mono-deuterated benzene molecule in the molecular plane for vibrational states corresponding to the first excitations of the normal modes shown to the left.

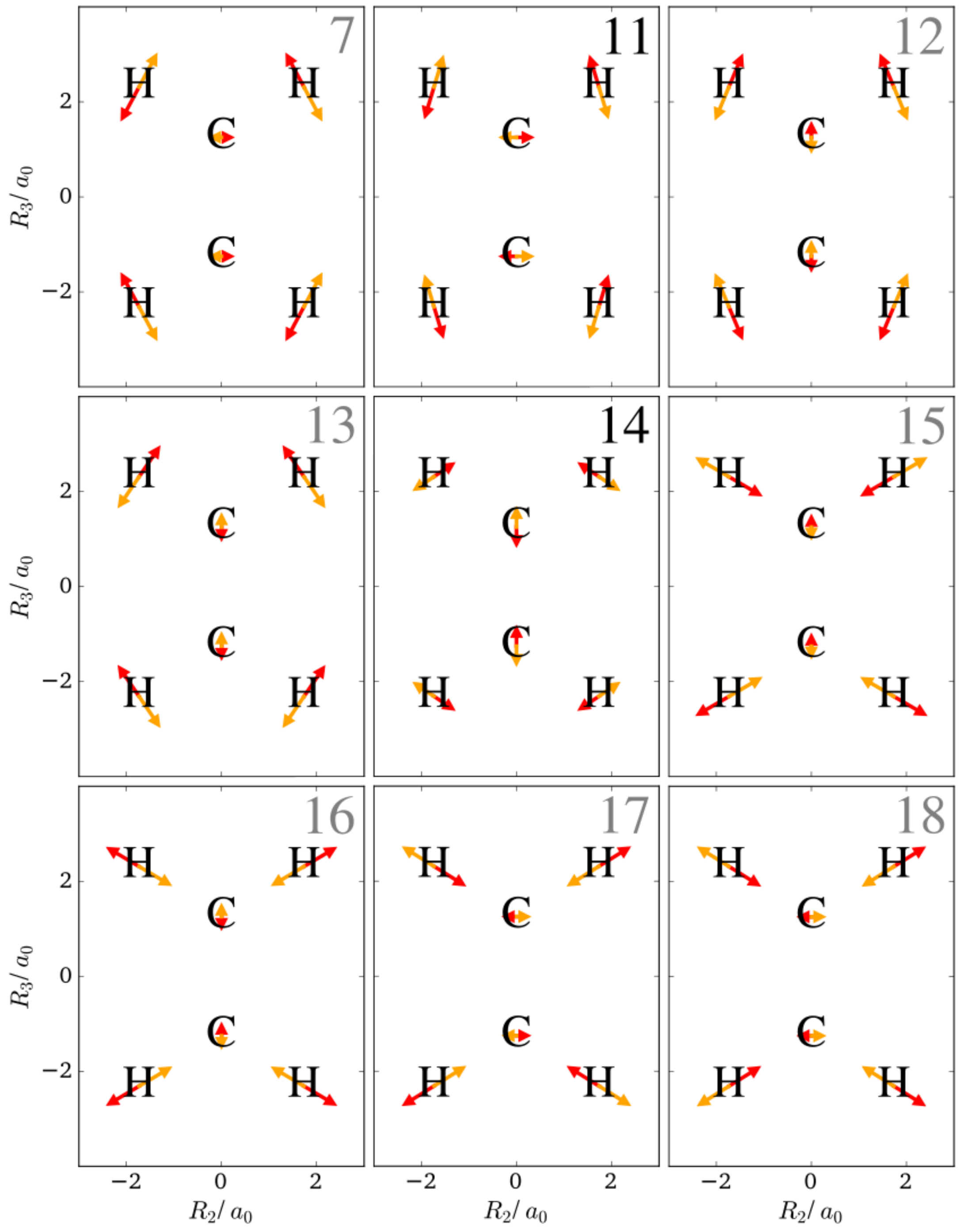

Figure 2: Normal modes of ethene that are confined to the molecular plane. The modes are labeled according to frequency, with modes 1-6 representing translation and rotation of the whole molecule. The arrows show the extent (length) of the displacement of the nuclei along the mode and the directionality of the displacement (color).

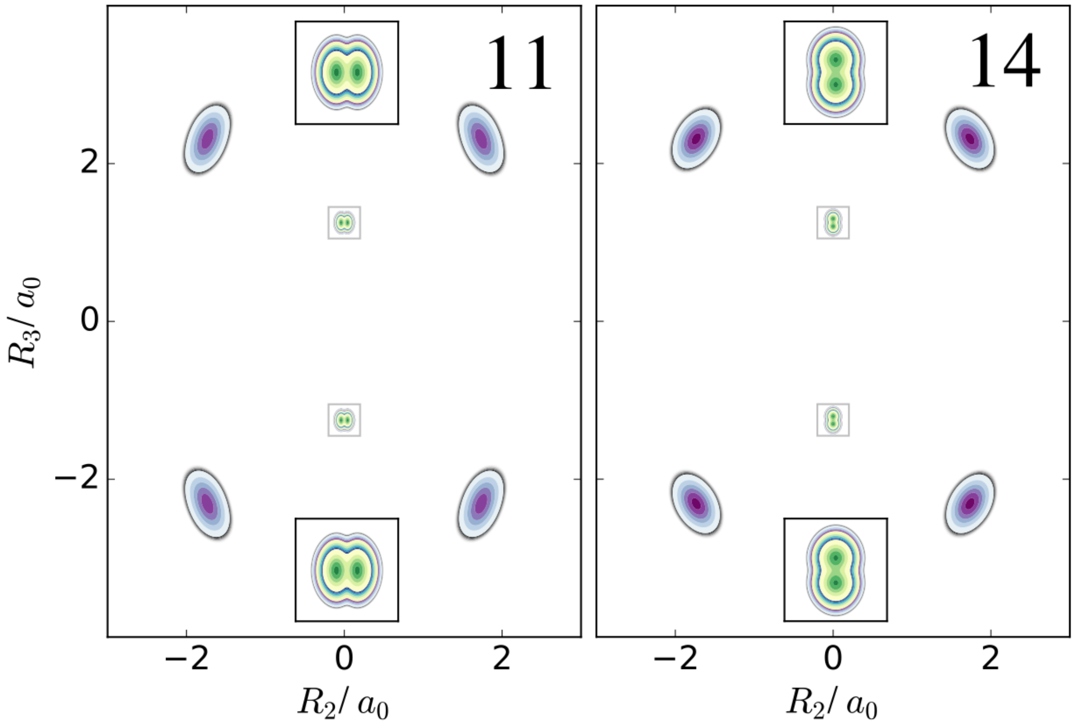

Figure 3: Contour plots of the one-nucleus densities of localized and oriented ethene in the molecular plane for the vibrational states corresponding to the first excitation along normal modes 11 and 14. Insets at the top and bottom show a magnified view of the region around the oxygen nuclei.

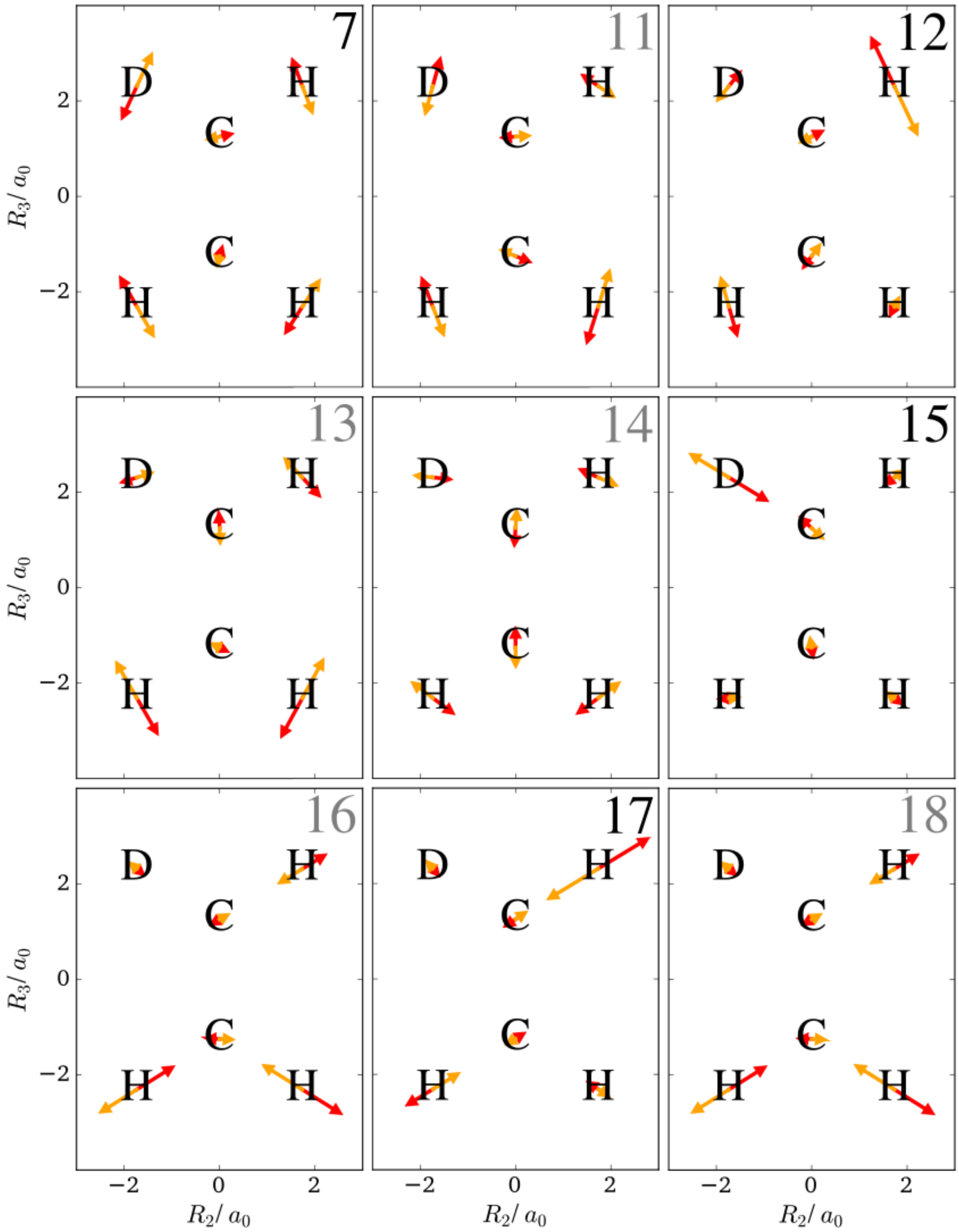

Figure 4: Normal modes of mono-deuterated ethene that are confined to the molecular plane. The modes are labeled according to frequency, with modes 1-6 representing translation and rotation of the whole molecule.

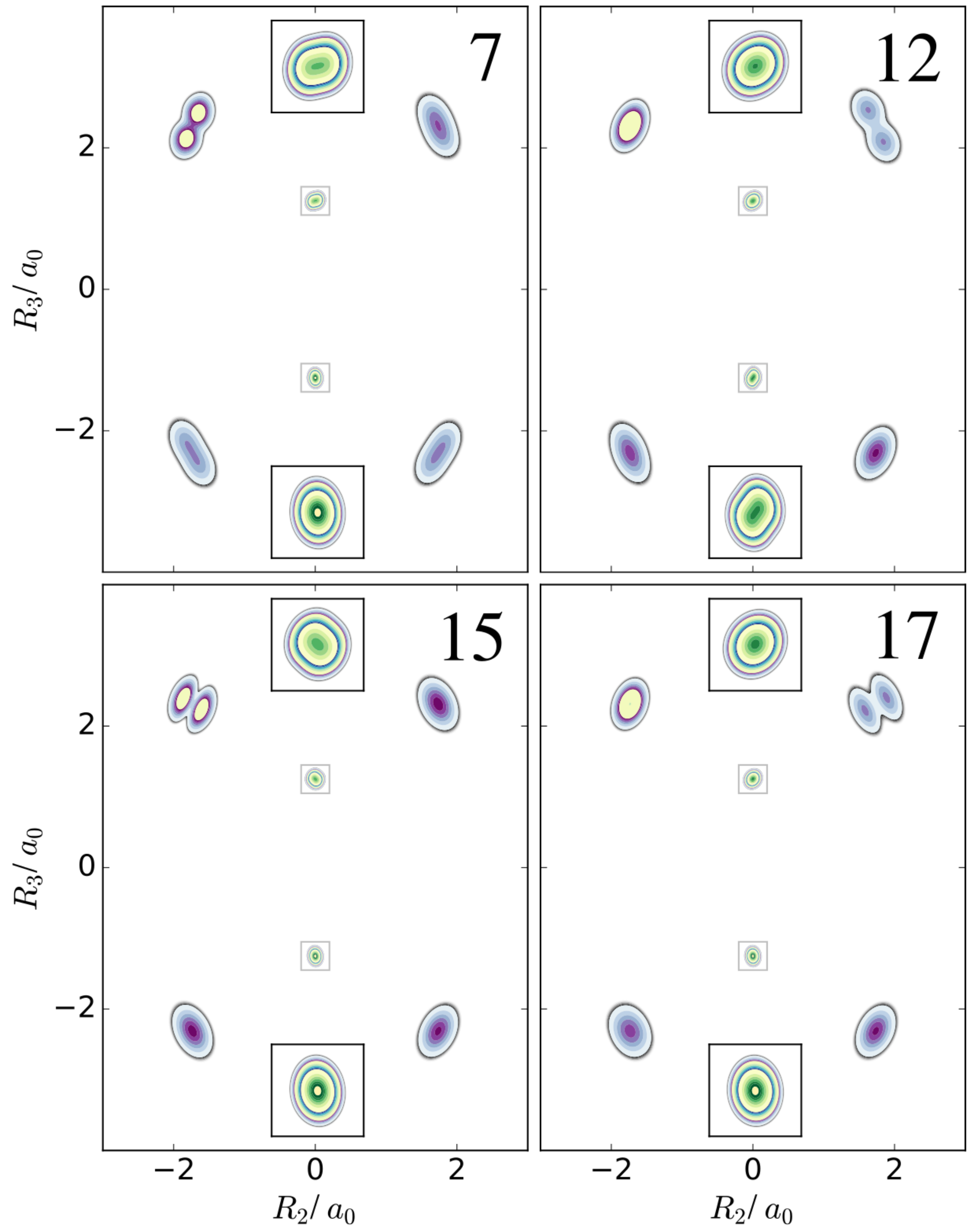

Figure 5: Contour plots of the one-nucleus densities of localized and oriented mono-deuterated ethene in the molecular plane for the vibrational states corresponding to the first excitation along normal modes 7, 12, 15, and 17. Insets at the top and bottom show a magnified view of the region around the oxygen nuclei.

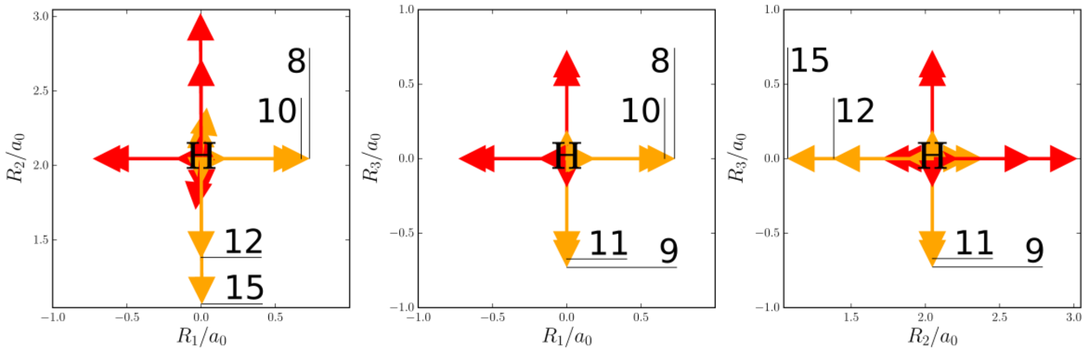

Figure 6: Normal modes of one of the hydrogen nuclei of methane in the R_1-R_2-, R_1-R_3-, and R_2-R_3-plane (the center of mass of the molecule is at the origin). For those corresponding to the largest displacements in a given direction, their numbers (ordered according to increasing frequency of the normal modes, with modes 1-6 corresponding to translation and rotation of the molecule) are given.

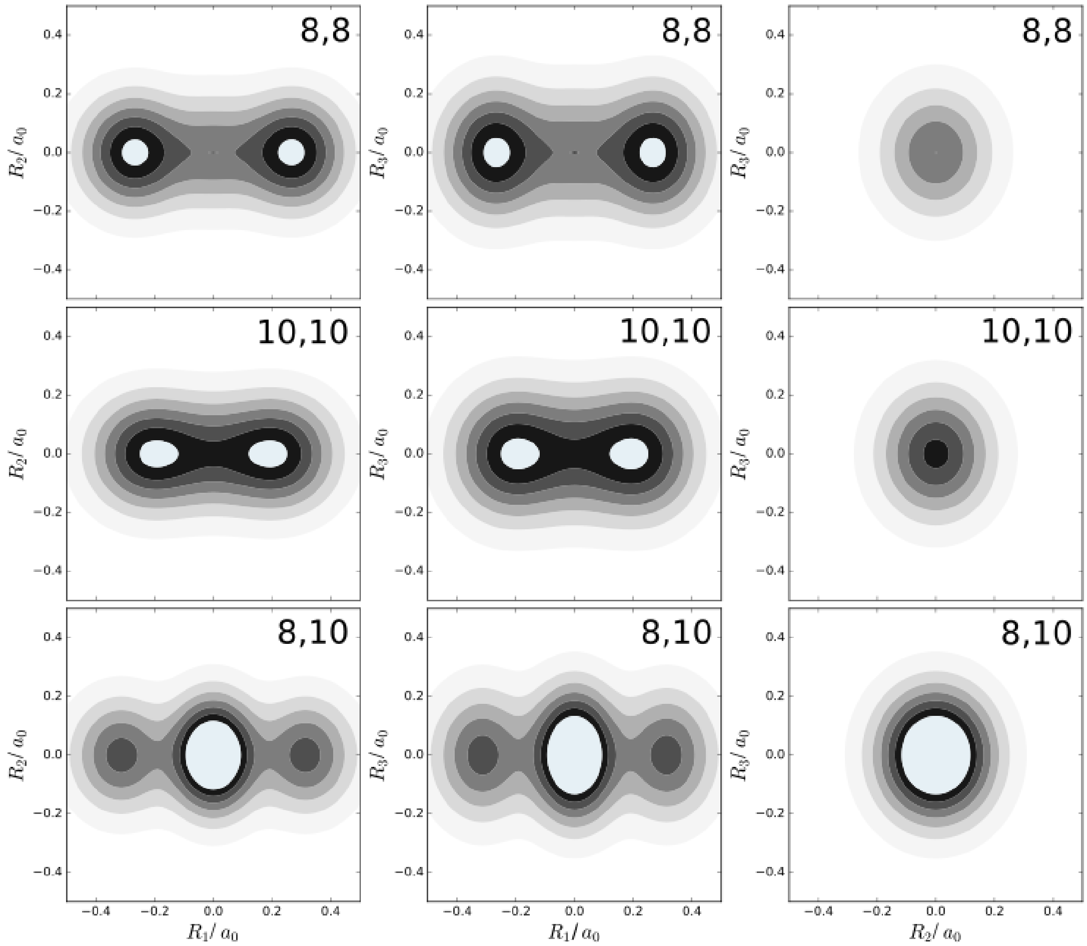

Figure 7: Contour plots of the one-nucleus densities of the hydrogen nucleus of methane of figure 6 for a state where two quanta are distributed in the normal modes. Left column: R_1-R_2-plane. Middle column: R_1-R_3-plane. Right column: R_2-R_3-plane. Top row: One-nucleus density for the state corresponding to the second excitation of mode 8. Middle row: One-nucleus density for the state corresponding to the second excitation of mode 10. Bottom row: One-nucleus density for the state corresponding to the first excitation of both mode 8 and mode 10. Note that compared to the normal modes in figure 6, the hydrogen nucleus was shifted to the origin in R_2-direction.

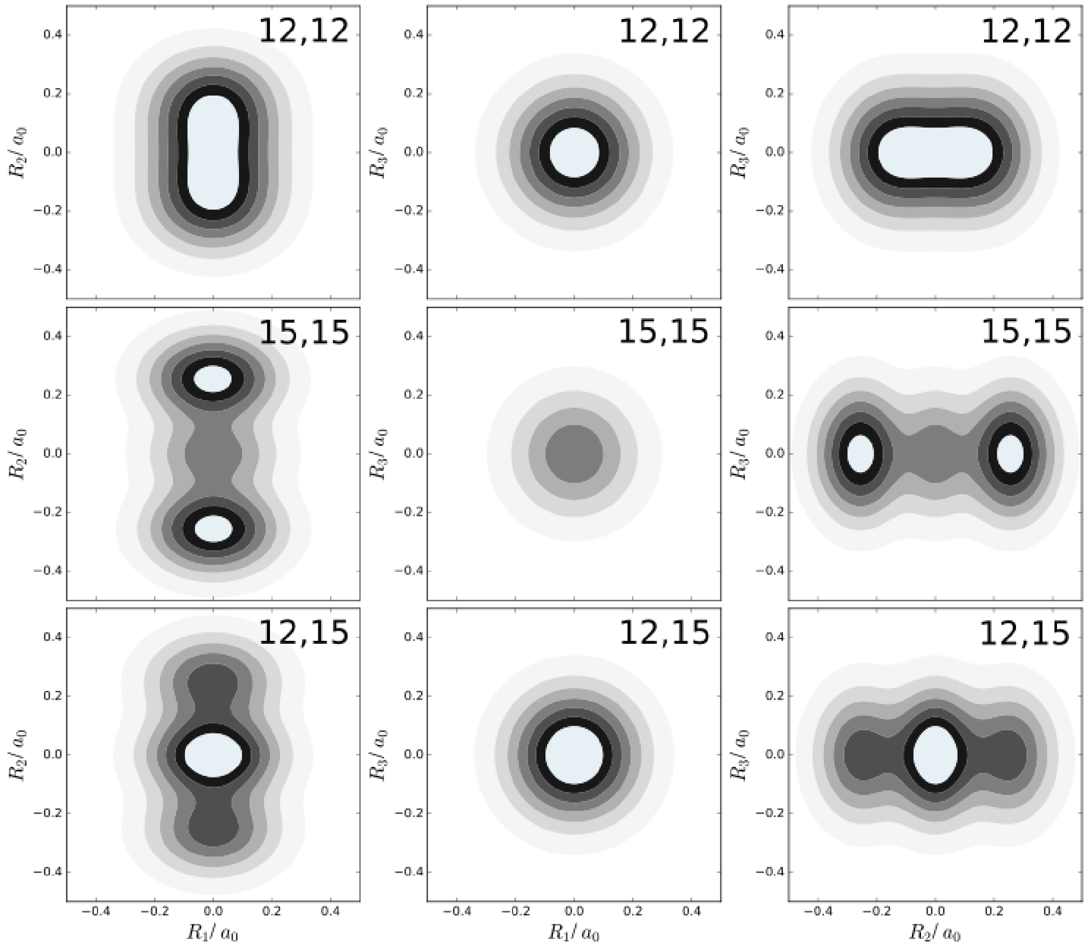

Figure 8: Contour plots of the one-nucleus densities of the hydrogen nucleus of methane of figure 6 for a state where two quanta are distributed in the normal modes. Left column: R_1-R_2-plane. Middle column: R_1-R_3-plane. Right column: R_2-R_3-plane. Top row: One-nucleus density for the state corresponding to the second excitation of mode 12. Middle row: One-nucleus density for the state corresponding to the second excitation of mode 15. Bottom row: One-nucleus density for the state corresponding to the first excitation of both mode 12 and mode 15. Note that compared to the normal modes in figure 6, the hydrogen nucleus was shifted to the origin in R_2-direction.

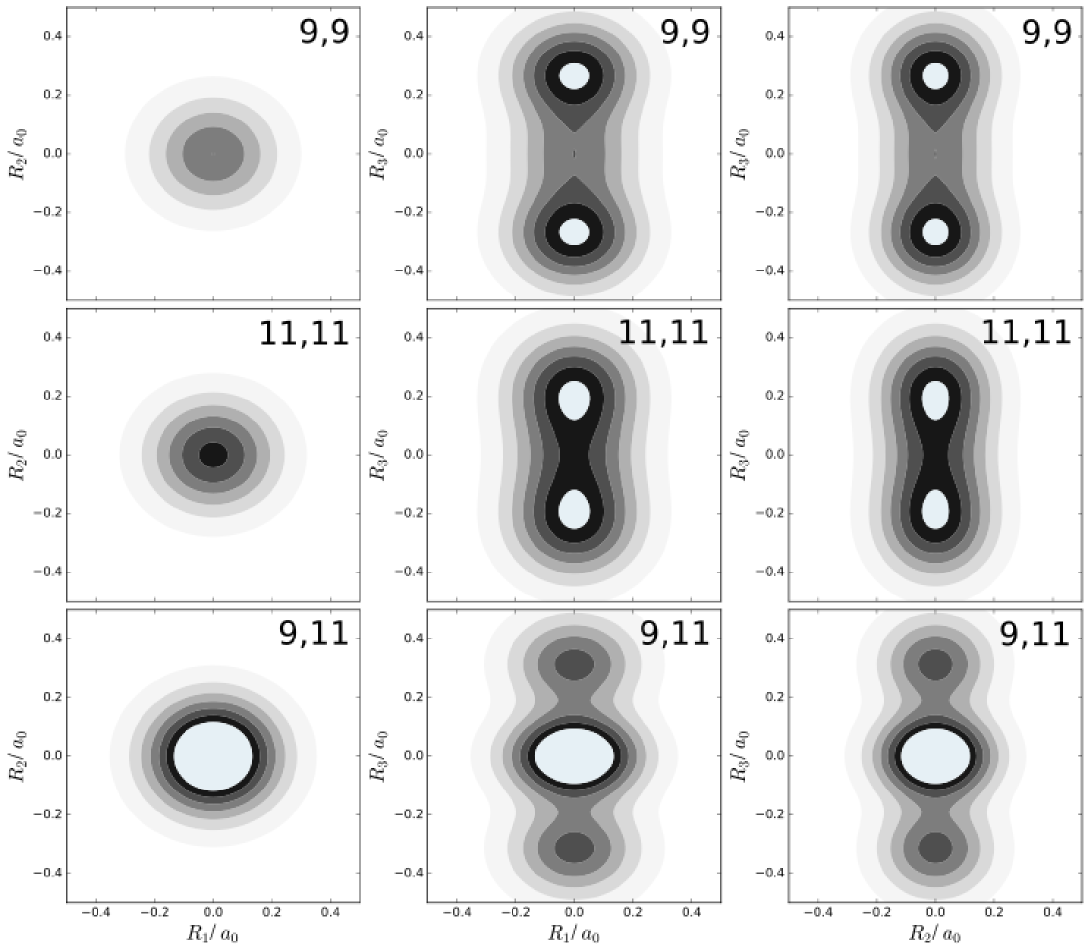

Figure 9: Contour plots of the one-nucleus densities of the hydrogen nucleus of methane of figure 6 for a state where two quanta are distributed in the normal modes. Left column: R_1-R_2-plane. Middle column: R_1-R_3-plane. Right column: R_2-R_3-plane. Top row: One-nucleus density for the state corresponding to the second excitation of mode 9. Middle row: One-nucleus density for the state corresponding to the second excitation of mode 11. Bottom row: One-nucleus density for the state corresponding to the first excitation of both mode 9 and mode 11. Note that compared to the normal modes in figure 6, the hydrogen nucleus was shifted to the origin in R_2-direction.
