# Supplementary material for: On the Probability Density of the Nuclei in a Vibrationally Excited Molecule
Source: Front Chem. 2019 Jun 6;7:424. doi: 10.3389/fchem.2019.00424 (PMC6562893; doi:10.3389/fchem.2019.00424)
Supplement: Supplementary file 2 [file Data_Sheet_2.PDF]

Supporting Information for "On the Probability Density of the Nuclei in a Vibrationally Excited Molecule": Computational Details

Axel Schild

March 1, 2019

1 The local harmonic approximation

The local harmonic approximation is briefly reviewed. For a detailed description, see [1]. In the Born-Oppenheimer approximation, the nuclear wavefunction is obtained from

$$\left(-\sum_{j=1}^{N}\frac{\hbar^2\partial_{X_j}^2}{2M_j}+V(X)\right)\Psi(X)=E\Psi(X) \tag{1}$$

with masses M_j for the coordinates X_j. Around a minimum X_{eq} of V, the potential is expanded in a Taylor series to second order in terms of the displacement coordinates $x = X - X_{eq}$ and $V(X_{eq})$ is set to zero. Additionally, mass-weighted displacement coordinates $\tilde{x}_j = \sqrt{M_j}x_j$ are introduced. Then (1) becomes

$$\left(-\sum_{j=1}^{N}\frac{\hbar^2\partial_{\tilde{x}_j}^2}{2}+\frac{1}{2}\sum_{j=1}^{N}\sum_{k=1}^{N}U_{jk}\tilde{x}_j\tilde{x}_k\right)\Psi(X(\tilde{x}))=E\Psi(X(\tilde{x})) \tag{2}$$

with

$$U_{jk}=\frac{\partial_{X_j}\partial_{X_k}V(X)\big|_{X=X_{eq}}}{\sqrt{M_jM_k}} \tag{3}$$

The matrix U is diagonalized by the matrix of eigenvectors Q,

$$Q^T\cdot U\cdot Q=\Omega \tag{4}$$

where $Q^TQ = QQ^T = \mathrm{diag}(1)$ and $\Omega = \mathrm{diag}(\omega^2)$ is the diagonal matrix of the eigenvalues ω_j^2. This yields

$$\sum_{j=1}^{N}\sum_{k=1}^{N}U_{jk}\tilde{x}_j\tilde{x}_k=\tilde{x}^T\cdot U\cdot\tilde{x}=\tilde{x}^T\cdot Q\cdot Q^TU\cdot Q\cdot Q^T\cdot\tilde{x}=\sum_{j=1}^{N}\omega_j^2\tilde{q}_j^2 \tag{5}$$

with normal mode coordinates

$$\tilde{q}_j=Q^T\tilde{x}=\sum_{k=1}^{N}Q_{kj}\tilde{x}_k=\sum_{k=1}^{N}\sqrt{M_k}Q_{kj}x_k. \tag{6}$$

In terms of the normal mode coordinates, (1) becomes

$$\left(\sum_{j=1}^{N}\left(-\frac{\hbar^2\partial_{\tilde{q}_j}^2}{2}+\frac{\omega_j^2}{2}\tilde{q}_j^2\right)\right)\Psi(X(\tilde{q}))=E\Psi(X(\tilde{q})) \tag{7}$$

With the coordinate scaling

$$q_j=\sqrt{\frac{\omega_j}{\hbar}}\tilde{q}_j=\sum_{k=1}^{N}\sqrt{\frac{\omega_jM_k}{\hbar}}Q_{kj}x_k \tag{8}$$

the wavefunction becomes a product

$$\Psi(X(q)):=\psi(q)=\prod_{j=1}^{N}\phi_j(q_j,m_j) \tag{9}$$

with the eigenfunctions of the quantum harmonic oscillator with unit mass and unit frequency[2, 3]

$$\phi_j(q_j, m_j) = \sqrt{2^{m_j} m_j!} \left(\frac{1}{\pi}\right)^{\frac{1}{4}} e^{-\frac{q_j^2}{2}} \sum_{k=0}^{\left\lfloor \frac{m_j}{2} \right\rfloor} \frac{(-1)^k}{4^k k!(m_j - 2k)!} q_j^{m_j - 2k}, \tag{10}$$

where the floor function $\lfloor r \rfloor$ gives the largest integer smaller than or equal to r. However, there is one catch: Six (or five, for linear molecules) of the frequencies ω_j are zero, as they belong to translations and rotations of the full system. There are no external potentials that break the translational and rotational invariance of the nuclear system, hence it is broken artificially by setting a non-zero value for these frequencies ω_j. The quantum numbers m_j for these degrees of freedom are set to zero so that the density in these modes is a Gaussian function with a width determined by the chosen frequency. In the limit $\omega_j \to 0$, a δ-distribution is obtained for $|\phi_j(q_j, 0)|^2$.

2 The density

In the main article, the nuclear one-nucleus density is denoted as $\rho(\mathbf{R})$ and it is defined as sum of the individual one-nucleus densities $\rho_j(\mathbf{R})$. In this notes it is described how to obtain $\rho_j(\mathbf{R})$, but the notation is slightly different. We start from the density $\rho^{[N]}(x_1, \dots, x_N)$ in the N-dimensional configuration space in terms of displacement coordinates x_j, and we aim at computing the one-nucleus density $\rho^{[3]}(x_1, x_2, x_3)$ of the particle with displacement coordinates x_1, x_2, x_3, by integrating over $x_4, \dots, x_N$. To obtain $\rho(\mathbf{R})$, all we need to do is to shift $\rho^{[3]}(x_1, x_2, x_3)$ to the equilibrium position of the considered nucleus, to repeat the procedure for the coordinates of all other nuclei, and to add all those densities. We use the notation $\rho^{[N]}$ because we give the solution of the integrals iteratively, by computing $\rho^{[N-1]}$, $\rho^{[N-2]}$, etc., with each of the densities in this series depending on one coordinate less compared to the previous density.

The explicit expression for the N_n-body or N-coordinate density $\rho^{[N]}(x)$ can now be given. We require that

$$\int \cdots \int |\Psi(X)|^2 dX_1 \dots dX_N = \int \cdots \int \rho^{[N]}(x) dX_1 \dots dX_N \stackrel{!}{=} 1 \tag{11}$$

and have

$$\int |\phi_j(q_j, m_j)|^2 dq_j = 1, \tag{12}$$

where $\int$ represents the definite integral $\int_{-\infty}^{\infty}$ throughout this text. Thus, the density is

$$\rho^{[N]}(x) = J_{qx} \prod_{j=1}^{N} \left(\phi_j(q_j, m_j)\right)^2 \tag{13}$$

with the Jacobian determinant J_{qx} for the coordinate transformation (8) from x to q that ensures that $\rho^{[N]}(x)$ is normalized to one when integrating over all x. Explicitly, with the transformation matrix

$$T_{jk} = \sqrt{\frac{\omega_j M_k}{\hbar}} Q_{kj} \tag{14}$$

we have

$$J_{qx} = |\det(T_{jk})| \tag{15}$$

Then

$$\rho^{[N]}(x) = J_{qx} \prod_{j=1}^{N} \frac{2^{m_j} m_j!}{\sqrt{\pi}} e^{-q_j^2} \sum_{k=0}^{\left\lfloor \frac{m_j}{2} \right\rfloor} \sum_{l=0}^{\left\lfloor \frac{m_j}{2} \right\rfloor} \frac{(-1)^{k+l}}{4^{k+l} k! l! (m_j - 2k)!(m_j - 2l)!} q^{2(m_j - (k+l))} \tag{16}$$

$$= J_{qx} \Gamma^{[N]}(x) \prod_{j=1}^{N} P_j^{(2m_j)}(x) \tag{17}$$

with the Gaussian function*

$$\Gamma^{[N]}(x) = \exp\left(-\sum_{j=1}^{N} q_j(x)^2\right) \tag{18}$$

*A factor $\pi^{N/2}$ could be added to have $\Gamma^{[N]}(x)$ properly normalized. Instead, it is included in the definition of the polynomial coefficients.

and with the polynomial of order $2m_j$

$$P_j^{(2m_j)}(x) = \frac{2^{m_j} m_j!}{\sqrt{\pi}} \sum_{k=0}^{\lfloor \frac{m_j}{2} \rfloor} \sum_{l=0}^{\lfloor \frac{m_j}{2} \rfloor} \frac{(-1)^{k+l}}{4^{k+l} k! l! (m_j - 2k)! (m_j - 2l)!} q(x)^{2(m_j - (k+l))} \tag{19}$$

Before inserting the explicit definition of $q_j(x)$, we rewrite this polynomial by changing the summation variables to $k + l \to k$, $(k - l)/2 \to l$, so that

$$P_j^{(2m_j)}(x) = \sum_{k=0}^{2\lfloor \frac{m_j}{2} \rfloor} c_{m_j,k} q(x)^{2(m_j - k)} = \sum_{k=0}^{2\lfloor \frac{m_j}{2} \rfloor} p_j^{(2(m_j - k))} \tag{20}$$

with coefficients

$$c_{m_j,k} = \frac{2^{m_j - 2k} m_j!}{\sqrt{\pi}} \sum_{l=-L_k}^{L_k} \frac{(-1)^k}{\left(\frac{k}{2} + l\right)! \left(\frac{k}{2} - l\right)! \left(m_j - 2\left(\frac{k}{2} + l\right)\right)! \left(m_j - 2\left(\frac{k}{2} - l\right)\right)!} \tag{21}$$

that are obtained with summation boundaries

$$L_k = \frac{1}{2}\left(\left\lfloor \frac{m_j}{2} \right\rfloor - \left| k - \left\lfloor \frac{m_j}{2} \right\rfloor \right| \right) \tag{22}$$

An alternative that is more practical for numerical implementations is to change the summation variables to $k + l \to k$, $(k - l)/2 \to l$, so that the coefficients become

$$c_{m_j,k} = \frac{2^{m_j - 2k} m_j!}{\sqrt{\pi}} \sum_{\substack{l=-L_k \\ 2|l}}^{L_k} \frac{(-1)^k}{\left(\frac{k+l}{2}\right)! \left(\frac{k-l}{2}\right)! \left(m_j - (k + l)\right)! \left(m_j - (k - l)\right)!} \tag{23}$$

$$L_k = \left\lfloor \frac{m_j}{2} \right\rfloor - \left| k - \left\lfloor \frac{m_j}{2} \right\rfloor \right| \tag{24}$$

where the sum over l now has increments $\Delta l = 2$.

3 Initial parameters

The general form of the N-nucleus density (17) is

$$\rho^{[N]}(x) = \Gamma^{[N]}(x) \left(A^{[N](0)} + \sum_{k_1,k_2=1}^{N} A^{[N](2)}_{k_1 k_2} x_{k_1} x_{k_2} + \cdots + \sum_{k_1,\ldots,k_{2M}=1}^{N} A^{[N](2M)}_{k_1 \ldots k_{2M}} x_{k_1} \ldots x_{k_{2M}} \right) \tag{25}$$

with the Gaussian function (18) given as

$$\Gamma^{[N]}(x) = \exp\left(-\sum_{j=1}^{N} \sum_{k=1}^{N} S^{[N]}_{jk} x_j x_k \right) \tag{26}$$

It is a multivariate Gaussian function multiplied by a polynomial composed of monomials of degree $0, 2, \ldots, 2M$, where

$$M = \sum_{j=1}^{N} m_j \tag{27}$$

is the sum of quantum numbers. The superscript $[N]$ of the coefficients for the monomials $A^{[N](2\alpha)}$ and $S^{[N]}_{jk}$ indicates that those are the parameters of the N-nucleus density. Below, we see that when we integrate over coordinate x_N we obtain an $N-1$-nucleus density $\rho^{[N-1]}$ that looks like (25), except that the sums terminate at $N-1$ and the coefficients changed. By determining the new coefficients, we can perform all integrals that are necessary to derive the one-nucleus density iteratively. Note that the Jacobian determinant is included in the initial coefficients, cf. (32).

However, first we have to determine the initial values of the coefficients. Inserting the transformation equation of the coordinates (8),(14) into the definition of the Gaussian function (18) yields

$$S^{[N]}_{jk} = \sum_{l=1}^{N} T_{lj} T_{lk} \tag{28}$$

Similarly, inserting (8),(14) into the definition of the polynomials (20) for each quantum number j shows that these polynomials are a sum (over k_j) of monomials

$$p_j^{2(m_j-k_j)} = J_{qx} \sum_{l_1=1}^{N} \cdots \sum_{l_{2(m_j-k_j)}=1}^{N} A^{(2(m_j-k_j)),j}_{l_1 \dots l_{2(m_j-k_j)}} x_{l_1} \dots x_{l_{2(m_j-k_j)}} \tag{29}$$

with coefficients†

$$A^{(2(m_j-k_j)),j}_{l_1 \dots l_{2(m_j-k_j)}} = c_{m_j,k_j} T_{jl_1} \dots T_{jl_{2(m_j-k_j)}} \tag{30}$$

The polynomial occurring in the definition of the density (17) is

$$\prod_{j=1}^{N} P_j^{(2m_j)} = \sum_{k_1=0}^{2\lfloor \frac{m_1}{2} \rfloor} \cdots \sum_{k_N=0}^{2\lfloor \frac{m_N}{2} \rfloor} p_1^{2(m_1-k_1)} \dots p_N^{2(m_N-k_N)} \tag{31}$$

By comparing the definition of the coefficients in the density (25) with the from of the polynomial (31) we see how to obtain the coefficients $A^{[N](2\alpha)}$: First, we compute all monomial coefficients (30). Then, we take the tensor product along j,‡

$$J_{qx} \times A^{(2(m_1-k_1)),1}_{l_1 \dots l_{2(m_1-k_1)}} \otimes A^{(2(m_2-k_2)),2}_{l_1 \dots l_{2(m_2-k_2)}} \otimes \cdots \otimes A^{(2(m_N-k_N)),N}_{l_1 \dots l_{2(m_N-k_N)}} \tag{32}$$

for all possible combinations of the k_j-index. The number of indices of the resulting object is the sum of the number of indices of the individual coefficients and corresponds to the order of the polynomial (2α) to which it belongs. Adding all the results of the same order yields the initial coefficients $A^{[N](2\alpha)}$ of the N-nucleus density.

4 Integration

Next, we need to integrate over one variable, say, x_N. For this purpose, we need the integral§ [3]

$$\int e^{-ax^2+bx+c} dx = \sqrt{\frac{\pi}{a}} e^{\frac{b^2}{4a}+c} = I_0 \tag{33}$$

Taking the derivative of (33) w.r.t. b and comparing with the definition of the Hermite polynomials yields

$$\int x^n e^{-ax^2+bx+c} dx = \sum_{m=0}^{\lfloor \frac{n}{2} \rfloor} \frac{n!}{2^n m!(n-2m)!} \frac{b^{n-2m}}{a^{n-m}} I_0 \tag{34}$$

Integrating the density (25) over x_M yields

$$\rho^{[N-1]}(x) = \int \rho^{[N]}(x) = \sum_{i=0}^{M} \sum_{j=0}^{2i} \sum_{k_1=1}^{N-1} \cdots \sum_{k_{i-j}=1}^{N-1} \hat{P}_j^{(2i)} A^{[N](2i)}_{k_1 \dots k_{2i-j} N \dots} x_{k_1} \dots x_{k_{2i-j}} I_j^{[N]} \tag{35}$$

Here, $A^{[N](2i)}_{k_1 \dots k_{2i-j} N \dots}$ represents the coefficient for the monomial of order $2i$ with $2i-j$ indices that run from 1 to $N-1$, and the remaining j indices set to N. The operator $\hat{P}_j^{(2i)}$ constructs the sum of all $\binom{2i}{j}$ permutations of the indices set to N with those that run from 1 to $N-1$.

† These coefficients are symmetric w.r.t. exchange of any two indices.

‡ These coefficients are not symmetric w.r.t. exchange of any two indices anymore, but only within certain blocks. This makes the equations later a little bit more elaborate.

§ $\int$ is still the definite integral from $x=-\infty$ to $x=\infty$.

To perform the integral of the density over the last coordinate $\mathtt{x_N}$, we first have to group the monomials in (25) for each order according to the exponent of $\mathtt{x_N}$ (which corresponds to the index $\mathtt{j}$ of $\mathtt{I_j}$) to find

$$\begin{aligned}\int \rho^{[N]}(x) =& A^{[N](0)} I_0 + \sum_{k_1=1}^{N-1}\sum_{k_2=1}^{N-1} A^{[N](2)}_{k_1k_2} x_{k_1} x_{k_2} I_0 + \sum_{k_1=1}^{N-1}\left(A^{[N](2)}_{k_1N} + A^{[N](2)}_{Nk_1}\right) x_{k_1} I_1 + A^{[N](2)}_{NN} I_2 \\ &+ \sum_{k_1=1}^{N-1}\sum_{k_2=1}^{N-1}\sum_{k_3=1}^{N-1}\sum_{k_4=1}^{N-1} A^{[N](4)}_{k_1k_2k_3k_4} x_{k_1}x_{k_2}x_{k_3}x_{k_4} I_0 \\ &+ \sum_{k_1=1}^{N-1}\sum_{k_2=1}^{N-1}\sum_{k_3=1}^{N-1}\left(A^{[N](4)}_{k_1k_2k_3N} + A^{[N](4)}_{k_1k_2Nk_3} + A^{[N](4)}_{k_1Nk_2k_3} + A^{[N](4)}_{Nk_1k_2k_3}\right) x_{k_1}x_{k_2}x_{k_3} I_1 \\ &+ \sum_{k_1=1}^{N-1}\sum_{k_2=1}^{N-1}\left(A^{[N](4)}_{k_1k_2NN} + A^{[N](4)}_{k_1Nk_2N} + A^{[N](4)}_{Nk_1k_2N} + A^{[N](4)}_{k_1NNk_2} + A^{[N](4)}_{Nk_1Nk_2} + A^{[N](4)}_{NNk_1k_2}\right) x_{k_1}x_{k_2} I_2 \\ &+ \sum_{k_1=1}^{N-1}\left(A^{[N](4)}_{k_1NNN} + A^{[N](4)}_{Nk_1NN} + A^{[N](4)}_{NNk_1N} + A^{[N](4)}_{NNNk_1}\right) x_{k_1} I_3 + A^{[N](4)}_{NNNN} I_4 + \dots \end{aligned} \tag{36}$$

At each order $\mathtt{2i}$, the coefficients of each integral $\mathtt{I_j}$ are obtained as sum of the $\binom{2i}{j}$ possible permutations of the index $\mathtt{N}$ occurring $\mathtt{j}$ times in the coefficients $A^{[N](2i)}$. Unfortunately, in general $A^{[N](2i)}$ is not symmetric when exchanging two indices. However, it is constructed as tensor product of arrays that have this property, hence this symmetry may be exploited to some extent, if desired.

We now assume (correctly) that $\rho^{[N-1]}$ has the same functional form as $\rho^{[N]}$, but with new coefficients $A^{[N-1]}$ and $S^{[N-1]}$,

$$\rho^{[N-1]}(x) \stackrel{!}{=} \exp\left(-\sum_{j=1}^{N-1}\sum_{k=1}^{N-1} S^{[N-1]}_{jk} x_j x_k\right)\left(A^{[N-1](0)} + \sum_{k_1,k_2=1}^{N-1} A^{[N-1](2)}_{k_1k_2} x_{k_1}x_{k_2} + \dots + \sum_{k_1,\dots,k_{2M}=1}^{N-1} A^{[N-1](2M)}_{k_1\dots k_{2M}} x_{k_1}\dots x_{k_{2M}}\right) \tag{37}$$

The integrals $I^{[N]}_j$ that occur here are of the form (34) and are given by

$$I^{[N]}_0 = \sqrt{\frac{\pi}{S^{[N]}_{NN}}}\exp\left(-\sum_{j=1}^{N-1}\sum_{k=1}^{N-1}\left(S^{[N]}_{jk} - \frac{S^{[N]}_{jN}S^{[N]}_{kN}}{S^{[N]}_{NN}}\right) x_j x_k\right) \tag{38}$$

and

$$I^{[N]}_n = \sum_{m=0}^{\lfloor \frac{n}{2}\rfloor} \frac{(-1)^n n!}{4^m m!(n-2m)!}\frac{I^{[N]}_0}{\left(S^{[N]}_{NN}\right)^{n-m}} \sum_{k_1=1}^{N-1}\cdots\sum_{k_{n-2m}=1}^{N-1} S^{[N]}_{k_1N}\dots S^{[N]}_{k_{n-2m}N} x_{k_1}\dots x_{k_{n-2m}} \tag{39}$$

respectively.

Equations (38) and (39) can be derived from (33) and (34) by making the identifications

$$a = S^{[N]}_{NN} \qquad b = -2\sum_{j=1}^{N-1} S^{[N]}_{jN} x_j \qquad c = -\sum_{j=1}^{N-1}\sum_{k=1}^{N-1} S^{[N]}_{jk} x_j x_k \tag{40}$$

We see from (38) that because $I^{[N]}_0$ occurs in all integrals $I^{[N]}_n$, the new coefficients for the exponential are

$$S^{[N-1]}_{jk} = S^{[N]}_{jk} - \frac{S^{[N]}_{jN}S^{[N]}_{kN}}{S^{[N]}_{NN}} \tag{41}$$

The new coefficients for the polynomial are

$$A^{[N-1](2\alpha)}_{k_1\dots k_{2\alpha}} = \sqrt{\frac{\pi}{S^{[N]}_{NN}}}\sum_{i=\alpha}^{M}\sum_{j=2(i-\alpha)}^{2i}\frac{(-1)^j j!}{4^{i-\alpha}(i-\alpha)!(j-2(i-\alpha))!}\frac{\hat{P}^{(2i)}_j A^{[N](2i)}_{k_1\dots k_{2i-j}N\dots} S^{[N]}_{k_{2i-j+1}N}\cdots S^{[N]}_{k_{2\alpha}N}}{\left(S^{[N]}_{NN}\right)^{j+\alpha-i}} \tag{42}$$

Some remarks are in order: First, we note that the indices $k_1, \dots$ now only have $N-1$ entries. Second, the term $A^{[N](2i)}_{k_1\dots k_{2i-j}N\dots} S^{[N]}_{k_{2i-j+1}N}\dots S^{[N]}_{k_{2\alpha}N}$ of the last equation has to be read as follows: We take $A^{[N](2i)}_{k_1\dots k_{2i}}$ and set the last j indices equal to N. Then, we make a tensor multiplication with so many vectors $S^{[N]}_{k_jN}$ that the resulting object has 2α indices.

The second step of the integration of $\rho^{[N]}(x)$ over x_N is to group the resulting polynomial according to the orders of the monomials and add the respective contributions. The structure of $\rho^{[N-1]}(x)$ can be visualized as follows:

$$\binom{0}{0}[0] \oplus [0_0] \qquad \text{(i=0)}$$

$$\binom{2}{0}[2] \oplus [0_0] + \binom{2}{1}[1] \oplus [1_0] + \binom{2}{2}[0] \oplus [0_1, 2_0] \qquad \text{(i=1)}$$

$$\binom{4}{0}[4] \oplus [0_0] + \binom{4}{1}[3] \oplus [1_0] + \binom{4}{2}[2] \oplus [0_1, 2_0] + \binom{4}{3}[1] \oplus [1_1, 3_0] + \binom{4}{4}[0] \oplus [0_2, 2_1, 4_0] \qquad \text{(i=2)}$$

$$\binom{6}{0}[6] \oplus [0_0] + \binom{6}{1}[5] \oplus [1_0] + \binom{6}{2}[4] \oplus [0_1, 2_0] + \binom{6}{3}[3] \oplus [1_1, 3_0] + \binom{6}{4}[2] \oplus [2_2, 3_1, 4_0] + \binom{6}{5}[1] \oplus [1_2, 3_1, 5_0] + \binom{6}{6}[0] \oplus [0_3, 2_2, 4_1, 6_0] \qquad \text{(i=3)}$$

The binomial coefficients are a reminder of the permutations induced by the permutation operator $\hat{P}_j^{(2i)}$ acting on $A^{[N](2i)}$. The number $[i-j]$ left of $\oplus$ is the degree of the polynomial that is not included in the integration (as it does not contain the integration variable), and has the coefficient $A^{[N](2i)}$. The numbers $[a, b, \dots]$ are the degrees of the polynomials coming from the integral $I_{(j)}^{[N]}$. The $\oplus$ means that the orders have to be added, i.e. $[1] \oplus [1, 3]$ represents one monomial of order 2 and one monomial of order 4 in the final expression. The colors of the numbers right of $\oplus$ indicate the same order of the resulting monomial and the subscript indicates the number m of (39) from which the monomial is obtained. We note that all contributions to $A^{[N-1](0)}$ come from the terms for $m = i$, all contributions to $A^{[N-1](2)}$ come from the terms for $m = i-1$, etc. From this structure, (42) can be obtained as follows: First, we change the labels of the summation variables of the coordinates in (39) from $k_1, \dots, k_{j-2m}$ to $k_{2i-j+1}, \dots, k_{2(i-m)}$,

$$I_j^{[N]} = \sum_{m=0}^{\lfloor \frac{j}{2} \rfloor} \frac{(-1)^j j!}{4^m m!(j-2m)!} \frac{I_0^{[N]}}{\left(S_{NN}^{[N]}\right)^{j-m}} \sum_{k_{i-j+1}=1}^{N-1} \cdots \sum_{k_{i-2m}=1}^{N-1} S_{k_{2i-j+1}N}^{[N]} \dots S_{k_{2(i-m)}N}^{[N]} x_{k_{2i-j+1}} \dots x_{k_{2(i-m)}} \tag{43}$$

so that we can insert this formula directly into the equation for the density after first integration (35),

$$\rho^{[N-1]}(x) = \sum_{i=0}^{M} \sum_{j=0}^{2i} \sum_{m=0}^{\lfloor \frac{j}{2} \rfloor} \frac{(-1)^j j!}{4^m m!(j-2m)!} \sqrt{\frac{\pi}{S_{NN}^{[N]}}} \frac{\Gamma^{[N-1]}}{\left(S_{NN}^{[N]}\right)^{j-m}} \sum_{k_1=1}^{N-1} \cdots \sum_{k_{i-2m}=1}^{N-1} \hat{P}_j^{(2i)} A_{k_1 \dots k_{2i-j} N \dots}^{[N](2i)} S_{k_{2i-j+1}N}^{[N]} \dots S_{k_{2(i-m)}N}^{[N]} x_{k_1} \dots x_{k_{2(i-m)}} \tag{44}$$

$$\stackrel{!}{=} \Gamma^{[N-1]} \left(A^{[N-1](0)} + \sum_{k_1, k_2=1}^{N-1} A_{k_1 k_2}^{[N-1](2)} x_{k_1} x_{k_2} + \dots + \sum_{k_1, \dots, k_{2M}=1}^{N-1} A_{k_1 \dots k_{2M}}^{[N-1](2M)} x_{k_1} \dots x_{k_{2M}} \right) \tag{45}$$

Now we have to identify $A^{[N-1](2\alpha)}$ of (45) in (44) by setting $m = i - \alpha$ and by ensuring that the limits of the sums over i and j are adjusted accordingly.

To obtain the one-nucleus density, equations (41) and (42) need to be iterated until only three indices are left. Those belong to the displacement coordinates x_1, x_2, x_3. In order to obtain the one-nucleus density for the other nuclei, the procedure is repeated after appropriate permutation of the columns of transformation matrix T_{jk}.

Last, we note that that we could ignore all factors $1/\sqrt{\pi}$ in the original and updated coefficients because each integration cancels one of the of the initially N factors. Then we need to multiply the final one-nucleus density with $\pi^{-3/2}$ to obey the normalization condition.

5 Note on the computational implementation

The approximations that are used for the nuclear wavefunction allow to compute the vibrational one-nucleus densities for molecules with a relatively large number of nuclei N_n. However, the resulting polynomial is of order $2M$, where M is the sum of the vibrational quanta in the system. The current numerical implementation stores arrays of the polynomial coefficients that are of dimension N_n^{2M}, hence with the number of quanta M the memory limit is quickly reached, so that in practice only computations for $M \leq 4$ are possible on a modern workstation. With a different numerical implementation this problem can possibly be avoided, but for large M the local harmonic approximation that is made in the normal mode analysis is questionable anyway, hence this is not practical restriction.

References

[1] Niels Engholm Henriksen and Flemming Yssing Hansen. *Theories of Molecular Reaction Dynamics: The Microscopic Foundation of Chemical Kinetics (Oxford Graduate Texts)*. Oxford University Press, 2011.

[2] Claude Cohen-Tannoudji, Bernard Diu, and Frank Laloë. *Quantenmechanik 1/2*. de Gruyter, Berlin, 2007.

[3] Alan Jeffrey and Hui-Hui Dai. *Handbook of Mathematical Formulas and Integrals*. Elsevier, 2008.
